# Expression and Functional Role of Orphan Receptor GPR158 in Prostate Cancer Growth and Progression

**DOI:** 10.1371/journal.pone.0117758

**Published:** 2015-02-18

**Authors:** Nitin Patel, Tatsuo Itakura, Shinwu Jeong, Chun-Peng Liao, Pradip Roy-Burman, Ebrahim Zandi, Susan Groshen, Jacek Pinski, Gerhard A. Coetzee, Mitchell E. Gross, M. Elizabeth Fini

**Affiliations:** 1 Institute for Genetic Medicine, University of Southern California, Los Angeles, United States of America; 2 Center for Applied Molecular Medicine and University of Southern California Westside Prostate Cancer Center, Los Angeles, United States of America; 3 University of Southern California /Norris Comprehensive Cancer Center, Los Angeles, United States of America; 4 Department of Cell & Neurobiology, Keck School of Medicine, University of Southern California, Los Angeles, United States of America; 5 Department of Medicine, Keck School of Medicine, University of Southern California, Los Angeles, United States of America; 6 Department of Molecular Microbiology & Immunology, Keck School of Medicine, University of Southern California, Los Angeles, United States of America; 7 Department of Ophthalmology, Keck School of Medicine, University of Southern California, Los Angeles, United States of America; 8 Department of Pathology, Keck School of Medicine, University of Southern California, Los Angeles, United States of America; 9 Department of Preventive Medicine, Keck School of Medicine, University of Southern California, Los Angeles, United States of America; 10 Department of Urology, Keck School of Medicine, University of Southern California, Los Angeles, CA, 90089-9075, United States of America; Northern Institute for Cancer Research, UNITED KINGDOM

## Abstract

Prostate cancer (PCa) is the second-leading cause of cancer-related mortality, after lung cancer, in men from developed countries. In its early stages, primary tumor growth is dependent on androgens, thus generally can be controlled by androgen deprivation therapy (ADT). Eventually however, the disease progresses to castration-resistant prostate cancer (CRPC), a lethal form in need of more effective treatments. G-protein coupled receptors (GPCRs) comprise a large clan of cell surface proteins that have been implicated as therapeutic targets in PCa growth and progression. The findings reported here provide intriguing evidence of a role for the newly characterized glutamate family member GPR158 in PCa growth and progression. We found that GPR158 promotes PCa cell proliferation independent of androgen receptor (AR) functionality and that this requires its localization in the nucleus of the cell. This suggests that GPR158 acts by mechanisms different from other GPCRs. GPR158 expression is stimulated by androgens and GPR158 stimulates AR expression, implying a potential to sensitize tumors to low androgen conditions during ADT via a positive feedback loop. Further, we found GPR158 expression correlates with a neuroendocrine (NE) differentiation phenotype and promotes anchorage-independent colony formation implying a role for GPR158 in therapeutic progression and tumor formation. GPR158 expression was increased at the invading front of prostate tumors that formed in the genetically defined conditional *Pten* knockout mouse model, and co-localized with elevated AR expression in the cell nucleus. Kaplan-Meier analysis on a dataset from the Memorial Sloan Kettering cancer genome portal showed that increased GPR158 expression in tumors is associated with lower disease-free survival. Our findings strongly suggest that pharmaceuticals targeting GPR158 activities could represent a novel and innovative approach to the prevention and management of CRPC.

## Introduction

G-protein coupled receptors (GPCRs) comprise a large clan of cell surface proteins that perform diverse cellular functions. GPCRs sense information about the environment and typically transduce a signal into the cell by binding and activation of heterotrimeric G proteins upon extracellular ligand binding [1]. Members of this clan have been extensively exploited for drug discovery and a large fraction of currently used drugs in the market target GPCRs [2]. GPCRs are classified into seven families via phylogenetic analysis of their defining feature: the seven transmembrane (7TM) domain [1]. The GPCR glutamate family contains 7 orphan receptors, three belonging to the gamma-aminobutyric acid receptor branch: GPR156, GPR158, and GPR179 [3,4]. GPR179 was recently shown to be required for depolarizing bipolar cell function in the retina, and mutations cause autosomal-recessive complete congenital stationary night blindness [5,6]. Two very recent publications provide the first characterization of GPR158 [7,8]. The first identified GPR158 expression in retinal bipolar neurons and demonstrated an unusual role as a plasma membrane scaffold protein, functioning to inhibit signaling by GPCRs that couple with the inhibitory family of Galpha proteins by binding to Gbeta5 and recruiting members of the R7 family of GTPase Activating Proteins (GAPs) to the plasma membrane [7]. The second publication was from our laboratory [8]. We identified GPR158 expression in trabecular meshwork (TBM) cells in the eye’s aqueous outflow pathways and its role in regulation of cell barrier function, possibly contributing to the pathophysiology of steroid-induced ocular hypertension and glaucoma.

Searching for other possible roles for GPR158, we identified a published microarray study showing GPR158 as one of the genes upregulated in androgen ablation-resistant metastatic tumor as compared to primary prostate tumors [9]. Another gene expression microarray study showed down-regulation of GPR158 by withdrawal of estrogen in human estrogen-sensitive breast cancer cells, or by tamoxifen (anti-estrogen) treatment [10]. Perhaps not coincidentally, both cancer types involve altered response to steroid hormones. Prostate cancer (PCa) is the second-leading cause of cancer-related mortality after lung cancer in men from developed countries [11]. Initially, the proliferation of PCa cells depends on androgens, thus androgen-deprivation therapy (ADT) is the primary treatment for patients with locally advanced PCa. ADT provides remission of the disease in more than 90% of patients however the duration of response is typically 2–3 years. Despite ADT treatment, metastatic PCa and recurrent disease returns after failure of localized treatments [12], a process known as castration-resistant PCa (CRPC). Overall, metastatic CRPC patients have a median survival time of only 16–18 months and the disease remains incurable [13].

It is widely acknowledged that CRPC develops through multiple mechanisms including androgen receptor (AR)-dependent pathways such as AR amplification and overexpression [14]; local androgen synthesis [15]; altered expression of AR co-activator and co-repressor proteins; and AR-independent pathways such as alternative survival pathways [16]. Targeting the AR pathway has been shown to offer the most feasible approach for new therapeutic agents since AR remains active in CRPC [17]. Another possible therapeutic focus for PCa therapy is the neuroendocrine (NE) cell phenotype. This cell type occurs in scattered foci within prostatic adenocarcinoma, similar to its distribution within ductal epithelial cells of the normal prostate. However, the density of NE cells is often greater in prostate carcinomas than in normal tissue, and studies have shown that NE differentiation (NED) is enriched in high-grade and high-stage tumors, and closely associated with the emergence of CRPC [18–20]. Prostate epithelial cells are believed to transdifferentiate to NE cells, which are shown to be different from the minor population of NE cells present in normal prostate [18–20]. NE cells can promote the proliferation of adjacent adenocarcinoma cells in an androgen-starved condition, leading to CRPC [21], which is supported by the observations of higher proliferative index of proximal PCa cells to NE cells as compared to distal cancer cells [22,23]. Moreover, cell culture studies have established that neurohormones and neuropeptides secreted by NE cells can support androgen-independent growth of PCa cells [24,25]. However, the precise origin and the causative factors of NED remain unknown. Therefore, identification of novel genes and molecular mechanisms in connecting these multiple pathways responsible for pathogenesis of CRPC is critically needed for developing novel therapeutic approaches.

PCa is typically associated with genetic alterations involving androgen sensitivity and the AR [26]. In this report, we investigated the expression, molecular regulation, subcellular localization and functional role of GPR158 using a set of four human PCa cell lines with different alterations in the AR resulting in various degrees of androgen-responsiveness, androgen-sensitivity and AR expression. We combine this with a mouse PCa model and bioinformatics analysis of human PCa datasets. Our findings suggest that elevation of GPR158 expression is an important oncogenic event that stimulates PCa cell proliferation and progression and thus may represent an innovative therapeutic target.

## Materials and Methods

### Cell Culture

Primary human prostate epithelial cells (PHPECs) were purchased from the American Type Culture Collection (ATCC, Manassas, VA) and maintained, as per their instructions, using the prostate epithelial cell basal medium containing cell growth kit. Four human PCa cell lines maintained in one of our labs (Coetzee) were used in these studies: LNCaP, C4-2B, PC-3 and DU145, all obtained originally from the ATCC. LNCaP and C4-2B cells were cultured in complete RPMI medium, while DU145 and PC-3 cells were grown in DMEM medium (Gibco-BRL, Bethesda, MA), both containing 10% fetal calf serum (FCS) at 5% CO_2_ at 37°C. The cells were split every 3 days.

The LNCaP cell line was established from a human lymph node metastatic lesion of prostatic adenocarcinoma. LNCaP cells express the AR carrying the common T877A mutation in the ligand-binding domain, creating broad steroid binding specificity. They are androgen-responsive, displaying growth stimulation when treated with androgens, and they are also androgen-dependent, undergoing growth arrest upon withdrawal of androgens [27–29]. The C4-2B cells were derived from a bone metastasis that grew in nude mice after inoculation with the LNCaP-derived, castration-resistant C4-2 cells [30,31]. Like LNCaP cells, C4-2B cells are androgen-responsive. Consistent with this, they retain a functional AR, although it displays lower affinity for androgens than the AR in LNCaP cells [32]. However, unlike LNCaP cells, C4-2B cells are androgen-insensitive, because upon withdrawal of androgens they do not undergo growth arrest [33]. The PC-3 and DU145 cell lines were established from human prostatic adenocarcinoma, metastatic to brain or bone, respectively. The PC-3 and DU145 cell lines used in this study were previously characterized by one of our labs (Coetzee) [34]. Both lines are androgen-nonresponsive and androgen-insensitive. The PC-3^AR+^ sub-line employed in this study expresses low levels of the AR mRNA and protein. While this does not result in sufficient functional protein to confer androgen-responsiveness, ectopic AR expression consistently elicited a much greater transactivation response in this sub-line than in the PC-3^AR-^ sub-line. The DU145 cells are AR negative. We performed our own western blot to confirm the absence or presence of AR protein in these two lines (S1 Fig.).

Treatment of PHPEC, LNCaP and C4-2B cells with the androgen, dihydrotestosterone (DHT, 10nM), was carried out in culture media containing 10% charcoal-stripped serum (CSS) to deplete androgens. For androgen starvation studies, LNCaP, PC-3, and DU145 cells were grown in 10% CSS.

### Reagents and Oligonucleotide Primers

The reagents used in the study were purchased as follows: lipofectamine LTX with PLUS reagent (Invitrogen, Carlsbad, CA); primary antibodies to the AR, prostate specific antigen (PSA), neuron specific enolase (NSE), epidermal growth factor receptor (EGFR) and transcription factor Sp1, and horseradish peroxidase-conjugated secondary antibodies (Santa Cruz Biotechnology, Santa Cruz, CA); anti-intracellular domain (ICD) and anti-extracellular domain (ECD) GPR158 antibodies and anti-alpha-tubulin antibodies (Sigma-Aldrich Corp., St. Louis, MO); anti-beta-actin antibody (Abcam, Cambridge, UK); high-Fidelity DNA polymerase, Phusion (Finnzymes Inc.,Woburn, MA); oligonucleotides primers (Valuegene INC, San Diego, CA); charcoal stripped fetal bovine serum (Atlanta Biologicals Inc., Lawrenceville, GA). All other reagents were purchased from Sigma. **Table 1** provides the sequences of all oligonucleotides primers used in this study. For knockdown experiments, we used a pool of three custom designed siRNA oligonucleotides (GenePharma Co. Ltd, Shanghai, China), the activity of which we characterized previously [8].

**Table 1:** Oligonucleotide primers used in this study.

Gene	Method	Forward sequence	Reverse sequence
GPR158	RT-PCR	atattgctacagaagcatatgag	atatttccagttgggcatagag
AR	RT-PCR	caggaaagcgacttcaccgc	ctggggtggaaagtaatagtc
PSA	RT-PCR	ggcagcattgaaccagaggag	gcatgaacttggtcaccttctg
NSE	RT-PCR	ctcatcagctcaggtctctc	ccttacacacggccagagac
β-actin	RT-PCR	cattgccgacaggatgcaga	ctgatccacatctgctggaa
GAPDH	RT-PCR	aacctgccaagtacgatgacatc	gtagcccaggatgcccttga
GPR158:ΔC	PCR	Ttaattgctagcatgggagccatggcttacc	gcttaccggtggtggaatcaaaagcaacccaatggtgac

The abbreviations are: GPR158, G protein-coupled receptor 158; GAPDH, glyceraldehyde 3-phosphate dehydrogenase; PCR, polymerase chain reaction; RT-PCR, reverse transcriptase–polymerase chain reaction.

### Quantitative Real-Time Reverse Transcription-PCR (qRT-PCR)

Total RNA isolated from PCa cell lines with Aurum total RNA mini kit (Bio-Rad, Hercules, CA) was subjected to qRT-PCR analysis using iScript one-step RT-PCR kit with SYBR Green (Bio-Rad) on CFX96 Touch Real-Time PCR Detection System (Bio-Rad), according to the manufacturer’s instructions. Relative quantification values of genes of interest were calculated as 2^–ΔΔCt^ by the comparative Ct method, where ΔΔCt = (Ct target mRNA of treated sample- Ct reference gene of treated sample)—(Ct target mRNA of control sample-Ct reference gene of control sample). We used beta-actin or GAPDH as a reference gene. The primers used for the estimation of GPR158, AR, PSA and NSE expression are shown in Table 1.

### Western Blot Analysis

The whole cell lysates were prepared using radioimmunoprecipitation assay (RIPA) lysis buffer (50 mM Tris, 150 mM NaCl, 0.1% SDS, 0.5% Sodium Deoxycholate, 1% NP-40) spiked with a cocktail of protease inhibitors for 20 min, followed by centrifugation at 10,000g for 10 min. Supernatants were collected and proteins in the extracts were separated by SDS-PAGE without boiling the samples and transferred to PVDF membranes. Membranes were probed with primary antibody against protein of interest and were developed by chemiluminescence with reagents Luminol enhancer solution and peroxide solution (GE Healthcare UK limited, Buckinghamshire, UK) and images were captured with Fujifilm imaging system (LAS-4000; Fujifilm, Tokyo, Japan). Protein loading was monitored by stripping and reprobing of the membrane with beta-actin antibody.

### Expression Constructs

Three expression constructs (GPR158-pcDNA 3.1(+), GPR158-GFP and GPR158-NLS-M1+2-GFP) used for these studies were described previously [8]. We also generated another GPR158-GFP fusion construct, designated as GPR158:ΔC (AA 1–665), with the entire ICD deletion using appropriate primers listed in Table 1.

### Transient Transfection

The indicated cells were transfected with either expression plasmids or siRNA constructs or promoter reporter plasmids using Lipofectamine LTX reagent as per supplier’s instructions. Briefly, 1–2 × 10^5^ cells/well in 2 mL complete media were seeded in six-well plates and grown until 70–80% confluence. DNA (2 μg) was diluted in 500 μL Opti-MEM I + PLUS reagent (2 μL), incubated for 5 min, Lipofectamine LTX (4.5 μL) was added and incubated for 30 min. Cell medium was replaced with fresh 2 mL antibiotics free medium and the mixture was added, and incubated for 6 hrs. Following incubation, the complexes were replaced with complete growth medium. At 3 days post-transfection, the cells were processed for either qRT-PCR analysis or western blotting or cell counting.

### Lentivirus Production and Cell Transduction

GPR158 cDNA was sub-cloned from the pcDNA 3.1(+), expression vector described above, into the pSLIK lentiviral expression vector (Addgene, Cambridge, MA). The lentivirus production and infection was carried out as described [35]. Briefly, the viral packaging was performed in HEK 293T cells after co-transfection of the pSLIK-GPR158, two packaging plasmids pMDLg/pRRE and pRSVREV, and VSV G envelope plasmid using Lipofectamine 2000. The viruses were harvested 72 hrs after transfection, and the viral particles are concentrated. LNCaP cells were infected at a low MOI to ensure <30% infection frequency and the stable cells (Lenti-GPR158) were generated with hygromycin selection at 400 μg/mL for 4-wks. For induction of GPR158, the stable LNCaP cells were treated with 100 and 500 ng/mL of doxycycline (Dox) for 72 hrs. The LNCaP cells infected with viral particles generated with only pSLIK vector (Lenti-vector) were used as a negative control. Following 3-days of Dox induction, the cells were subjected to either western blotting or cell counting or subcellular protein fractionation assay or soft agar colony formation assay.

### Subcellular Fractionation

Subcellular fractionation was carried out using subcellular protein fractionation kit according to the manufacturers' instructions (Thermo Scientific Inc, Rockford, IL). This kit has been shown to isolate cytoplasmic, membrane, nuclear soluble, chromatin bound and cytoskeleton fractions of sufficient purity for protein localization and redistribution studies [36]. Briefly, a stepwise separation of cellular compartments from 3×10^6^ of indicated cells was performed after application of the supplied cytoplasmic, membrane, nuclear soluble, chromatin-bound and cytoskeletal protein extraction buffers. The protein concentration in each fraction was estimated using the BCA protein estimation kit (Thermo Scientific Inc.) and the indicated amount of protein was subjected to western blotting.

### Colony Formation Assay

The lenti-GPR158 or lenti-vector LNCaP cell lines were used for the colony formation assay. Briefly, the cells were plated in a 12-well plate (5,000 cells/well). The cells were suspended in phenol red free RPMI-1640 containing 10% FBS, and 0.48% agar, and overlaid onto a solid layer of the same medium containing 0.8% agar. The cells were either induced in the above medium containing 100 ng/mL Dox, or left untreated, with gentle replenishment of their media every 3 days. After a 2-week incubation, the plate was stained with 0.005% crystal violet (EMD Chemicals Inc, Gibbstown, NJ), destained overnight with PBS and colonies were counted under microscope and photographed at × 20 magnification.

### Immunohistochemical (IHC) Analysis in the Conditional *Pten* Knockout Mouse

The conditional *Pten* knockout mouse model was created at the University of Southern California by two of the co-authors of this study [37]. IHC analysis of GPR158 and AR expression was carried out in all three (ventral, dorsolateral and anterior) lobes derived from the prostate of a 10-month old conditional homozygous *Pten* knockout mouse (*Pten* ^-/-^) and a corresponding age-matched control (*Pten* ^*+/+*^). Briefly, the prostate tissues were fixed in 4% formaldehyde and the sample blocks were cut into 5 μm thick sections, deparaffinized in xylene, and rehydrated using a decreasing ethanol gradient followed by rinsing with double-distilled H_2_0. To retrieve antigenicity, slides were incubated with 10 mM citrate buffer (pH 6.0), washed with 0.1 M phosphate buffer, blocked with 0.3% hydrogen peroxide in methanol for 15 min and with 5% goat serum in PBS for 30 minutes at room temperature. The slides were incubated with anti-ICD GPR158 antibody (1:100) or anti-AR antibody (1:200) in 1% goat serum at 4°C overnight followed by incubation with biotinylated anti-rabbit secondary antibody (Vector Laboratories, Burlingame, CA) for 1 h and horseradish peroxidase streptavidin (Vector Laboratories) for 30 min, and then visualized by DAB kit (Vector Laboratories). The slides were subsequently counterstained with 5% (w/v) Harris Hematoxylin. The slides containing paraffin sections of the prostate from normal and PCa human subjects were provided by the core and processed using the above procedure.

### Statistical Analysis

Results are expressed as the mean ± standard error of the mean (S.E.) The significance of differences in mean values between untreated and treated samples was determined by the Student's t-test after determining that the data fit a normal distribution.

## Results

### GPR158 Effects on Cell Proliferation

**Fig 1:**
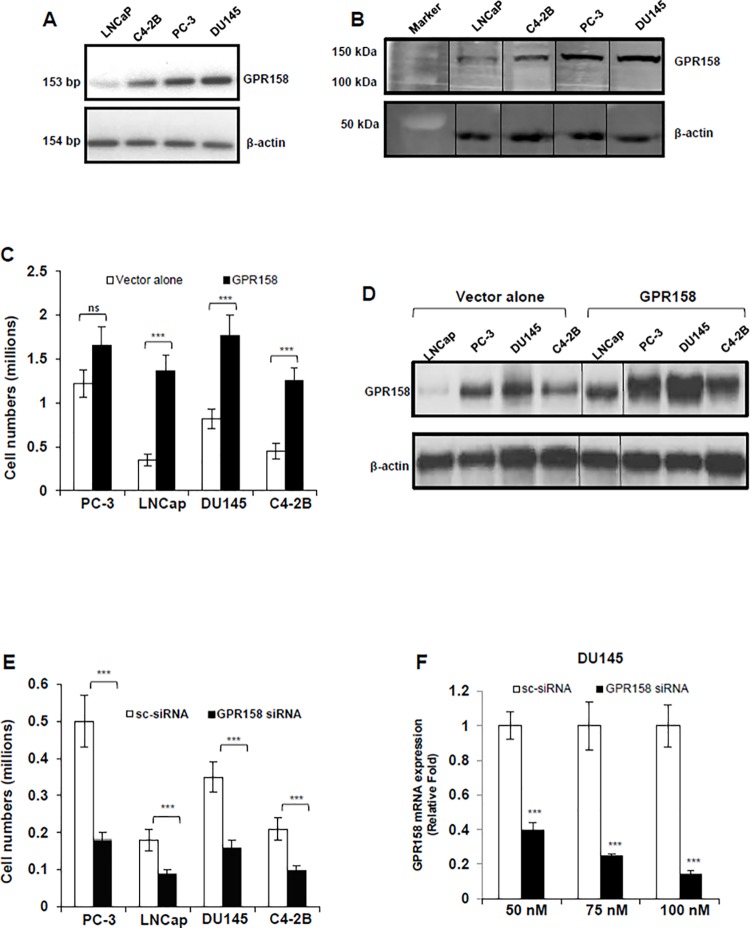
GPR158 effects on cell proliferation in PCa cell lines. **(A)** RT-PCR analysis of GPR158 mRNA expression in indicated PCa cell lines **(B)** Western blot analysis for the detection of GPR158 protein using anti-ICD GPR158 antibody in whole cell lysates of indicated PCa cell lines. The same membrane was probed for beta-actin, which served as a loading control. The vertical line indicates repositioned gel lanes from the same membrane to match the sample order of Fig. 1A. **(C, D, E)** All four indicated PCa cell lines were transfected at 70% confluence with either GPR158 expression plasmid or empty pcDNA3.1 (+) vector **(C and D)** OR either GPR158 siRNA or control scrambled siRNA **(E)** as indicated using Lipofectamine LTX reagent and incubated in growth medium for 3 days in a 6-well culture plates. **(C and E)** After 3 days, the trypsinized cells were counted using trypan blue dye in a hemocytometer chamber. **(D)** Western blotting for the detection of GPR158 in whole cell lysates isolated from cells transfected with indicated plasmids using anti-ICD GPR158 antibody. **(F)** Total RNA was isolated from DU145 cells that were transfected with the indicated concentration of either GPR158 siRNA or control scrambled siRNA for analyzing the levels of GPR158 mRNA by qRT-PCR. GAPDH was internal reference control. **(C, E, F)** The data shows mean ± SE of 3 independent experiments, each performed in duplicate. ***p<.001; **p<.01; *p<.05; ns, p>.05.

To investigate the significance of GPR158 expression to PCa, we performed a set of experiments using human PCa cell lines with different alterations in the AR resulting in various degrees of androgen-responsiveness, androgen-sensitivity and AR expression, as described in the Materials and Methods section. First we assessed the levels of endogenous GPR158 mRNA and protein by real-time quantitative PCR and western blotting, respectively. The rapidly-growing, androgen-insensitive lines DU145 and PC-3 (doubling time 1.5–2 days) expressed higher levels of GPR158 mRNA (Fig. 1A) and protein (Fig. 1B) as compared to slower growing cells of the LNCaP line and its derivative C4-2B (doubling time 2–3 days). The GPR158 protein level in the more rapidly-growing cells was approximately 2–3-fold higher as compared to more slowly-growing cells.

To evaluate the possible biological role of GPR158 in regulation of cell proliferation of human PCa cells, we performed transient transfections with the GPR158 mammalian expression vector previously described [8]. Cell lines transfected with vector alone were used as a control. The effect on cell proliferation was quantified after 3-days of transfection. Transient over-expression of GPR158 in cells of the LNCaP, C4-2B and DU145 lines led to a significantly higher rate of cell proliferation by 3.88, 2.15 and 2.77-fold, respectively as compared with cells transfected with the empty vector (Fig. 1C). Transient over-expression of GPR158 showed only a marginal increase in the proliferation of PC-3 cells, however, this was the most rapidly proliferating of the four cell lines without GPR158 over-expression, raising the possibility that the proliferation rate might already be maximal. The overexpression of GPR158 at 3 days post-transfection was confirmed in whole cell lysates by western blotting; a clear increase in GPR158 protein levels was observed in all of the cell lines (Fig. 1D).

To assess the role of endogenous GPR158 in PCa cell proliferation, we performed the converse experiment by down-regulation of GPR158 through the siRNA approach. We previously reported the characterization of a pool of three siRNA oligonucleotides, demonstrating 80–90% knockdown of GPR158 protein expression in PC-3 cells at 100 nM, in a reproducible manner [8]. We transfected each of the four PCa cell lines with 100 nM of this siRNA pool. After 3-days of transfection, significant growth inhibition (approximately 50%) was observed in all four of the cell lines, as compared with their respective scrambled siRNA transfected control cells (Fig. 1E). We confirmed the dose-dependent knockdown of GPR158 mRNA expression by transfection of the siRNA pool, with approximately 90% inhibition at 100 nM of siRNA in DU145 cells (Fig. 1F) and approximately 75–90% in the other cell lines (data not shown).

Together these results indicate that the expression level of endogenous GPR158 protein controls the rate of PCa cell proliferation, irrespective of the androgen-responsiveness, androgen-sensitivity or AR-expression status of the human PCa cell line used.

### Effects of an Androgen on GPR158 Expression

To discover factors regulating GPR158 expression, we searched the NextBio “Pharmaco Atlas” application [38] that finds compounds and treatments significantly correlated to a gene with results ranked in order of statistical significance. Our search using “GPR158” as the query identified the androgen, DHT as the most significant and the first in the list of correlated compounds. The results revealed 7 independent studies performed using LNCaP cells and all the studies showed increased GPR158 mRNA expression with DHT treatment in the range of 1.28–3.57-fold (S2 Fig.).

**Fig 2:**
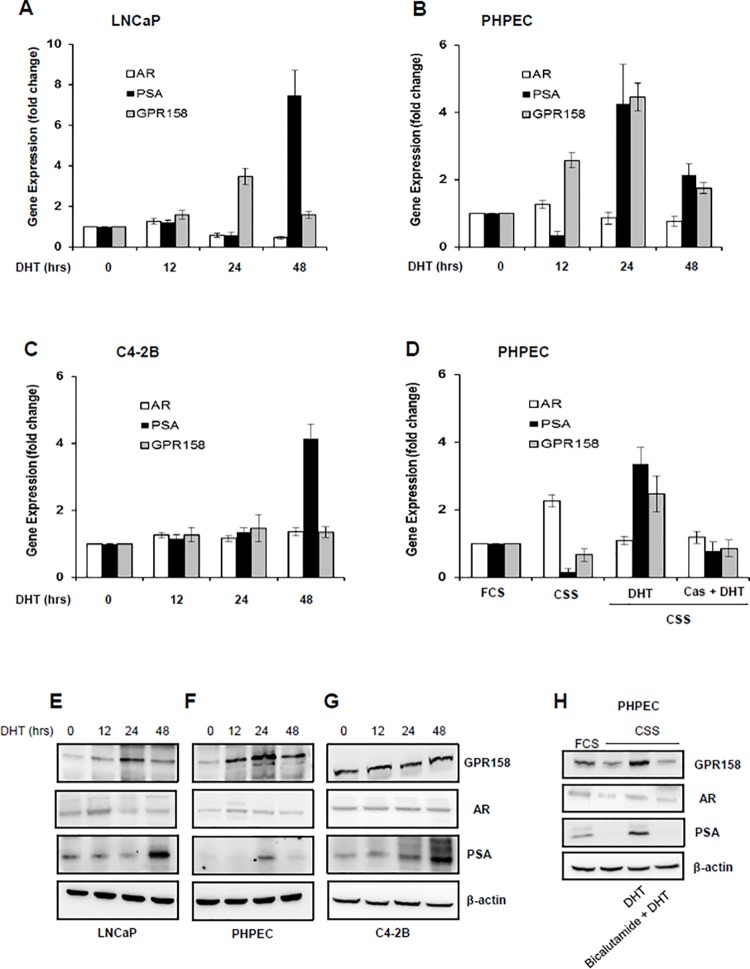
Effects of an androgen on GPR158 expression. **(A-C and E-G)** Cells of the indicated lines were incubated in media containing 10% CSS for androgen starvation overnight (0 time point), followed by treatment with DHT (10 nM) for the indicated time periods. Total RNA **(A-C)** or cell lysates **(E-G)** were isolated and subjected to qRT-PCR and western blotting, respectively to estimate AR, PSA, GPR158 and beta-actin expression. **(D and H)** PHPEC were grown in media containing 10% FCS or 10% CSS, treated with DHT (10nM) alone for 24 hrs or pre-incubated with bicalutamide (10μM) for 1-hr prior to DHT treatment in media containing 10% CSS. Total RNA or the whole cell lysates were isolated and qRT-PCR and western blotting was performed, respectively to detect GPR158, AR, PSA and beta-actin levels **(D)** The mRNA level of the above indicated genes was considered 1 in cells grown in 10% FCS for calculating the relative fold difference of these genes in other experimental conditions. **(A-H)** The data represent three independent experiments.

To determine experimentally whether GPR158 is an androgen-responsive gene, we carried out DHT treatment time course experiments, comparing androgen-responsive and androgen-sensitive PHPECs and LNCaP cells, and androgen-responsive, but androgen-*insensitive* C4-2B cells. Representative results are shown in Fig. 2. In PHPECs and LNCaP cells, GPR158 mRNA and protein levels increased by 3–4-fold with DHT treatment at 24 hrs (Fig. 2, A, B, E and F). In contrast, DHT treatment had no effect on GPR158 mRNA and protein levels in C4-2B cells (Fig. 2, C and G). Both mRNA and protein levels for prostate specific antigen (PSA), a well-studied AR target gene, increased significantly in PHPEC, LNCaP and C4-2B cells treated with DHT. Similar to published reports, we also observed AR protein stabilization in DHT-stimulated LNCaP cells at 12 hrs in PHPEC and LNCaP cells [39], but not in C4-2B cells (Fig. 2, E, F and G). Importantly, we found that the AR antagonist, bicalutamide inhibited the DHT-mediated increase in GPR158 mRNA and protein levels and we also observed a complete inhibition of PSA expression in PHPEC (Fig. 2, D and H). GPR158 mRNA and protein levels were reduced upon androgen starvation by incubation of PHPEC in media containing 10% CSS overnight. Based on these results, we conclude that GPR158 is an androgen-responsive gene.

### GPR158 Effects on AR Expression

**Fig 3:**
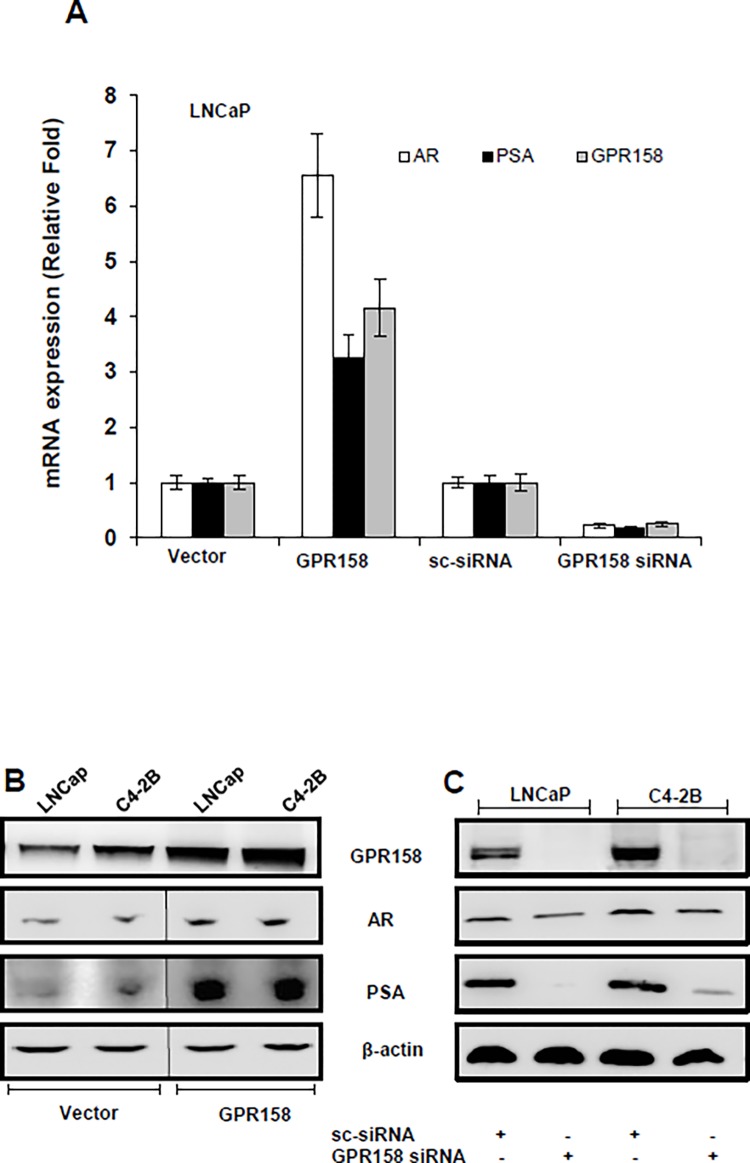
GPR158 effects on AR expression in the LNCaP/C4-2B model. LNCaP **(A, B, C)** and C4-2B **(B and C)** were transiently transfected using Lipofectamine LTX reagent with either GPR158 expression plasmid or vector **(A and B)** OR either GPR158 siRNA or control scrambled siRNA **(A and C)**. After 3 days of transfection, total isolated RNA and protein cell lysates were used for qRT-PCR **(A)** and western blotting **(B and C)**, respectively for the expression analysis of GPR158, AR, PSA and β-actin mRNA and protein. The data represent two independent experiments performed in duplicate.

The LNCaP/C4-2B human PCa progression model display characteristics of the transition from androgen-dependence to androgen insensitivity [32,40], and the AR plays a crucial role in this process [41]. Fig. 3 shows the results of our analysis of GPR158 effects on AR expression. Transient over-expression of GPR158 in LNCaP cells increased, and silencing of endogenous GPR158 reduced, mRNA for the AR, as well as for PSA (Fig. 3A). In both LNCaP and C4-2B cells, over-expression of GPR158 significantly increased AR and PSA protein levels, while knockdown of GPR158 (90% reduction) significantly reduced AR and PSA protein levels (Fig. 3, B and C). Together, these results indicate that GPR158 stimulates AR and PSA expression.

### Dose-Dependent Effects of GPR158

To further investigate the role of GPR158 in regulation of cell proliferation and AR gene expression, we created the lenti-GPR158 LNCaP cell line. This is a subline of LNCaP cells stably-transformed with a lentivirus that expresses GPR158 under the control of Dox. The Dox-inducible lentivirus system allows for tight regulation of GPR158 expression in a dose-dependent manner. LNCaP cells were chosen for this model because they express the lowest endogenous GPR158 levels. It was recently shown that Dox alters the metabolic and proliferation profiles of LNCaP cells at higher concentrations [42], an effect that we confirmed (data not shown). Thus, in all experiments with this cell line, we compare results obtained with the lenti-GPR158 LNCaP cell line to those obtained using parallel cultures of parental LNCaP cells treated with the same doses of Dox.

**Fig 4:**
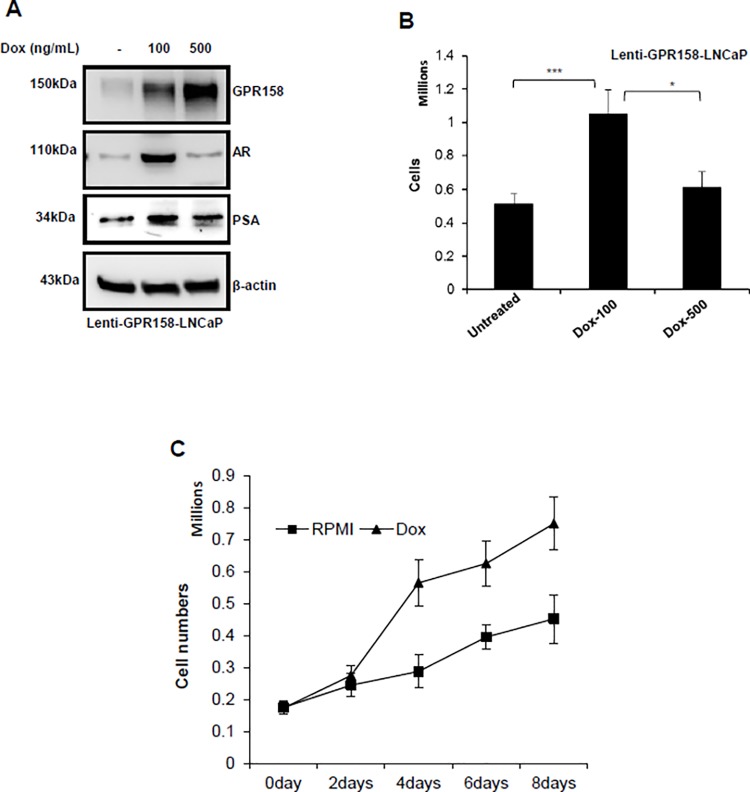
Dose-dependent effects of GPR158 in LNCaP cells. **(A and B)** LNCaP cells stably infected with lentivirus constructs containing GPR158 driven by a doxycycline inducible promoter were treated with low dose (100 ng/mL) or high dose (500 ng/mL) doxycycline concentration for 3-days. **(A)** The cell lysates were subjected to western blotting for the confirmation of induction of GPR158, AR, PSA and beta-actin proteins. **(B and C)** After doxycycline treatment for 3-days at indicated dose **(B)** and indicated time-points at 100ng/mL for growth curve **(C)**, the cells were trypsinized and counted using trypan blue dye in a hemocytometer chamber. The data represent normalized values with separate experiment performed to estimate the effect of doxycycline alone on parental LNCaP cell numbers under the same experimental conditions as above. **(B and C)** The results are indicative of three independent experiments, each performed in triplicate.

Fig. 4 shows representative results of experiments using the lenti-GPR158 LNCaP cell line. Treatment of lenti-GPR158 LNCaP with Dox for 3 days induced GPR158 expression in a dose-dependent manner, as compared to untreated cells (Fig. 4A). We consistently observed an approximately 3-fold and 6-fold increase in GPR158 protein levels after treating with Dox at 100 ng/mL and 500 ng/mL, respectively, for 3 days (Fig. 4A). A substantial increase in expression of AR and PSA was also observed with Dox treatment at 100 ng/mL, but interestingly, not with Dox treatment at 500 ng/mL (Fig. 4A). The membrane was re-probed for beta-actin as a loading control. The parental LNCaP cells treated with the same concentrations of Dox showed no change in the levels of GPR158, AR, PSA, or beta-actin in comparison with un-induced cells, indicating that the failure to induce AR and PSA is not due to Dox treatment at an elevated dose (S3 Fig.). In this same experiment, we also examined effects of GPR158 induction on cell proliferation. A 2.25-fold increase in cell numbers was observed with Dox treatment at 100 ng/mL as compared to controls, similar to results obtained with transient transfection (Fig. 4B). However, Dox at 500 ng/mL had only a small effect on cell proliferation (Fig. 4B) even though we normalized for the effects of Dox alone, on the parental LNCaP cells. Next, we performed growth curve analysis of stable lenti-GPR158 LNCaP cells and measured cell number over a period of 8-days post-Dox induction at 100 ng/mL. We found an approximately 2-fold increase in cell numbers at 4, 6 and 8-days under Dox treatment of lenti-GPR158 LNCaP, as compared to untreated cells (Fig. 4C). We confirmed the induction of GPR158 protein by 3-fold at 4, 6 and 8-days in Dox-treated cells (data not shown).

These results are consistent with those in Fig. 1 and 3, showing that GPR158 stimulates cell proliferation and AR expression in PCa cells. However, they further indicate that the effect of GPR158 is dose-dependent, with lower levels of GPR158 protein being stimulatory, but higher levels having less ability to stimulate cell proliferation and AR expression.

### GPR158 Nuclear Localization and Stimulation of Cell Proliferation

**Fig 5:**
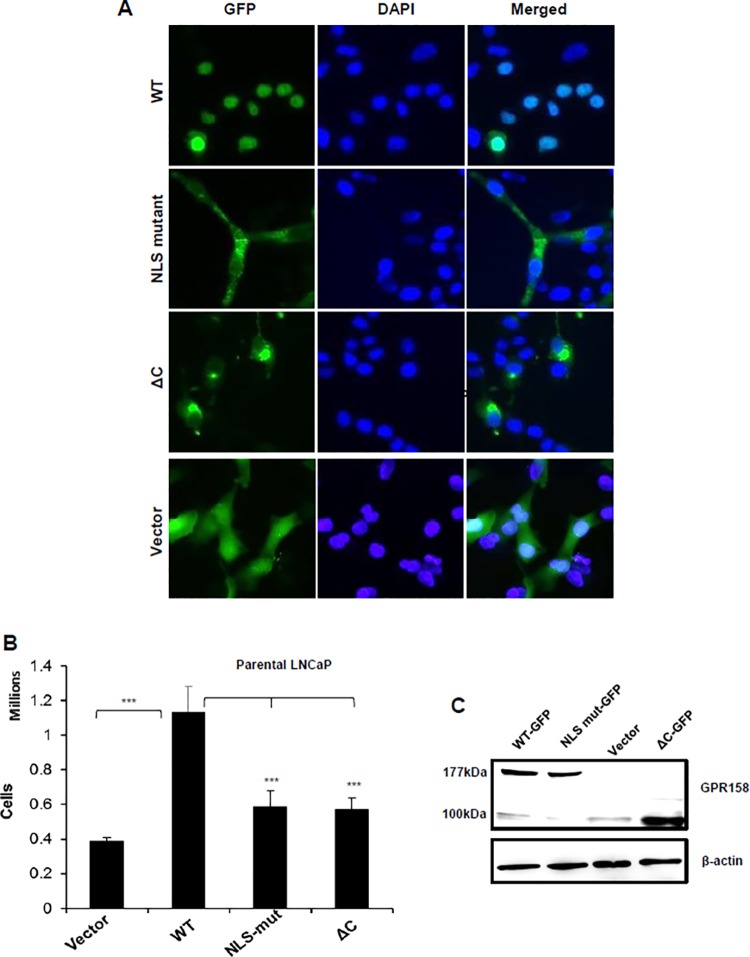
GPR158 nuclear localization and stimulation of cell proliferation in LNCaP cells. LNCaP cells were transiently transfected with GPR158, GPR158 NLS-double mutant or GPR158:ΔC expression constructs using Lipofectamine LTX reagent. Empty vector was used as control. **(A)** After 3 days of transfection, the cells were fixed and PBS washed, and then the slides were mounted using VECTASHIELD with DAPI and fluorescent images were captured using Nikon Eclipse Ti-E fluorescence microscope. The merged images show GFP as green and nuclear stain DAPI as blue. The images represent two independent experiments, each performed in triplicate. **(B)** The transfected cells were trypsinized and counted using trypan blue dye in a hemocytometer chamber. The data represent the mean ± SEM for three independent experiments, each performed in triplicate. **(C)** The cell lysates from the above mentioned transfected cells were subjected to western blotting for the detection of GPR158-GFP fusion protein using anti-ECD GPR158 antibody. The same membrane was striped and re-probed for beta-actin as a loading control. The data represent two independent experiments, each performed in triplicate.

We previously identified the presence of a bipartite nuclear localization signal (NLS) situated at the distal end of GPR158’s 8^th^ helix, spanning amino acids (AA) 718–732 in the ICD, and demonstrated its role in nuclear localization of GPR158 in TBM cells of the eye [8]. Here we further investigated the role of the NLS in LNCaP cells. To demonstrate subcellular localization, we used a previously created GPR158-GFP fusion construct with a double point mutation in the NLS [8]. We also generated a new construct designated as GPR158:ΔC (AA 1–665), in which the entire ICD was deleted. Each of these constructs was transiently transfected into LNCaP cells and GFP distribution examined by fluorescence microscopy. Representative results are shown in Fig. 5A. Wild-type GPR158-GFP was found predominantly in the nucleus of LNCaP cells. As expected, GFP fluorescence was distributed throughout the cells transfected with vector only, while both the NLS mutant and ΔC truncation proteins were localized primarily to the cytoplasm (Fig. 5A).

Transient over-expression of wild-type GPR158-GFP led to a 2.75-fold increase in cell proliferation, while the NLS double mutant construct and the ΔC construct failed to demonstrate any significant increase in cell proliferation (Fig. 5B). We confirmed that the levels of wild type, NLS mutant and ΔC-truncation proteins were similar in cells transfected with respective GFP-fusion constructs by western blotting using the anti-ECD GPR158 antibody (Fig. 5C). These results indicate that nuclear localization of GPR158 is required to stimulate cell proliferation.

### Subcellular Localization of Endogenous GPR158

**Fig 6:**
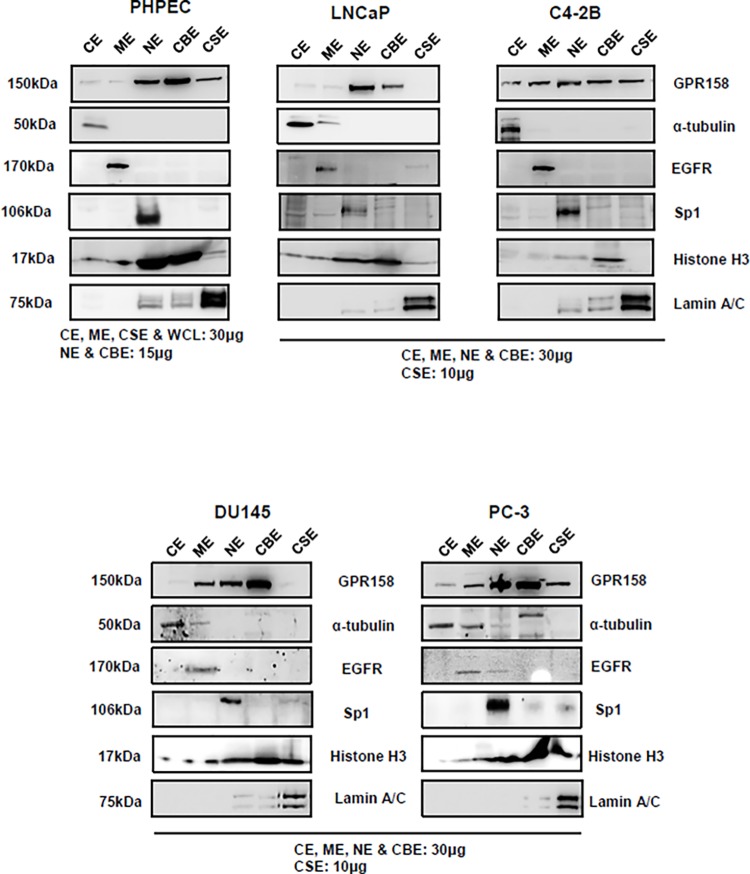
GPR158 subcellular localization in PCa cell lines and PHPECs. Subcellular protein fractionation was carried out in LNCaP, C4-2B, PC-3 and DU145 cells, as well as PHPECs. The amount of protein loaded for each fraction is indicated on the panels. Western blotting was performed using anti-ICD GPR158 antibody. Specific protein markers were used to validate and confirm the purity of the five subcellular fractions examined: cytoplasmic extract (CE) = alpha-tubulin, membrane extract (ME) = EGFR, soluble nuclear extract (NE) = Sp1, chromatin-bound nuclear extract (CBE) = histone H3 and insoluble cytoskeletal extract (CSE) lamin A/C. The percentage of GPR158 protein present in each fraction was calculated with respect to the total amount of protein in all fractions using Image J analysis of protein band intensities. The data are representative of two independent experiments.

To determine whether patterns of GPR158 subcellular localization might change with GPR158 expression levels in our four human PCa cell lines, we carried out biochemical fractionation followed by western blotting. For comparison, we also examined GPR158 subcellular localization in normal PHPEC. Representative results are shown in Fig. 6. Specific protein markers were used to validate and confirm the purity of the five subcellular fractions examined: cytoplasmic extract (CE), membrane extract (ME), soluble nuclear extract (NE), chromatin-bound nuclear extract (CBE) and insoluble cytoskeletal extract (CSE).

In the low GPR158-expressing LNCaP cells, ∼90–95% of the total GPR158 was localized to the soluble nuclear and chromatin bound nuclear fractions. GPR158 was strikingly redistributed across all the fractions in the relatively higher GPR158-expressing C4-2B cells as compared with LNCaP, with only ∼50% of the total protein localized to the soluble nuclear and chromatin bound nuclear fractions. A portion of GPR158 in this cell type was clearly present in the membrane fraction. This membrane localization was also seen in the two cell lines that express the highest levels of GPR158, PC-3 and DU145. C4-2B cells also exhibited clear localization of GPR158 to the cytoskeletal fraction in comparison to LNCaP cells. In PC-3, a portion of GPR158 was also localized to the cytoskeletal fraction. However cytoskeletal localization was not seen in DU145 cells.

In androgen-sensitive PHPECs, ∼85% of the total GPR158 was localized to the soluble nuclear and chromatin bound nuclear fractions, with greater localization to the latter, as in the rapidly growing PCa cell lines. Membrane localization was not noticeable, but there was some GPR158 presence in the cytoskeletal fraction. It is important to note that the PHPECs were cultured as specified by ATCC in a specific growth media, different from the media used for culture of the PCa cells lines, and its components could affect GPR158 subcellular distribution.

In conclusion, GPR158 is distributed in various subcellular fractions and different cell lines exhibit different subcellular distribution profiles. However the primary site of GPR158 localization is nuclear in all cases.

### GPR158 in NED

Using Oncomine, an online cancer-profiling database [43], we performed a search for expression microarray datasets that included GPR158. The search identified the Ma Breast 4 dataset. Analysis of this dataset for genes co-expressed with GPR158 revealed 28 candidates with a correlation of 0.5 or higher (Table 2). Of note, 20 of those genes are specific for neuronal and NE cells, including the classical NE markers chromogranin A and chromogranin B [44]. Also of interest in this list of 28 genes was RGS7, previously linked to GPR158 activity at the plasma membrane in the retina [7]. These results suggest that GPR158 might have a specific functional role in NE cells.

**Table 2:** Co-expression analysis of GPR158 in the Oncomine Ma Breast 4 dataset.

Gene	Correlation	NE Expression
CHGB (chromogranin B)	0.820	Yes
CARTPT (CART prepropeptide)	0.820	Yes
INA (internexin neuronal filament protein, alpha)	0.820	Yes
RAB3C	0.820	Yes
VSTM2A (V-set and transmembrane domain containing 2A)	0.820	Yes
CSMD3 (CUB and Sushi multiple domains 3)	0.820	
S100G (S100 calcium binding protein G)	0.820	Yes
SYT4 (synaptotagmin IV)_	0.820	Yes
LRTM2 (leucine-rich repeats and transmembrane domains 2)	0.820	
CGA (glycoprotein hormones, alpha polypeptide)	0.820	Yes
SCG2 (secretogranin II)	0.810	Yes
PCSK1 (proprotein convertase subtilisin/kexin type 1)	0.810	Yes
CHGA (chromogranin A)	0.795	Yes
EDDM3B (epididymal protein 3B)	0.770	
LOC90925 (hypothetical protein LOC90925)	0.747	
DDX25 (DEAD (Asp-Glu-Ala-Asp) box polypeptide 25)	0.702	Yes
RGS7 (regulator of G-protein signaling 7)	0.650	Yes
KCNJ3 (potassium inwardly-rectifying channel, subfamily J, member 3)	0.645	Yes
LINGO1 (leucine rich repeat and Ig domain containing 1)	0.610	Yes
CPB1 (carboxypeptidase B1)	0.556	Yes
USP45 (ubiquitin specific peptidase 45)	0.556	
CST9L (cystatin 9-like)	0.540	
CST9 (cystatin 9)	0.540	
PAH (phenylalanine hydroxylase)	0.540	
INSM1 (insulinoma-associated 1)	0.540	Yes
GP2 (glycoprotein 2)	0.540	Yes
SLC30A8 (solute carrier family 30 (zinc transporter), member 8)	0.529	Yes
GPR88	0.529	Yes

Using, GPR158 as a query, we searched the Oncomine database and the applied co-expression filter with the gene rank threshold by top 10%. We selected the genes with a correlation score of ≥ 0.5 and 20 out of 28 correlated genes were found to be expressed in NE cells of various organs as indicated in the table.

**Fig 7:**
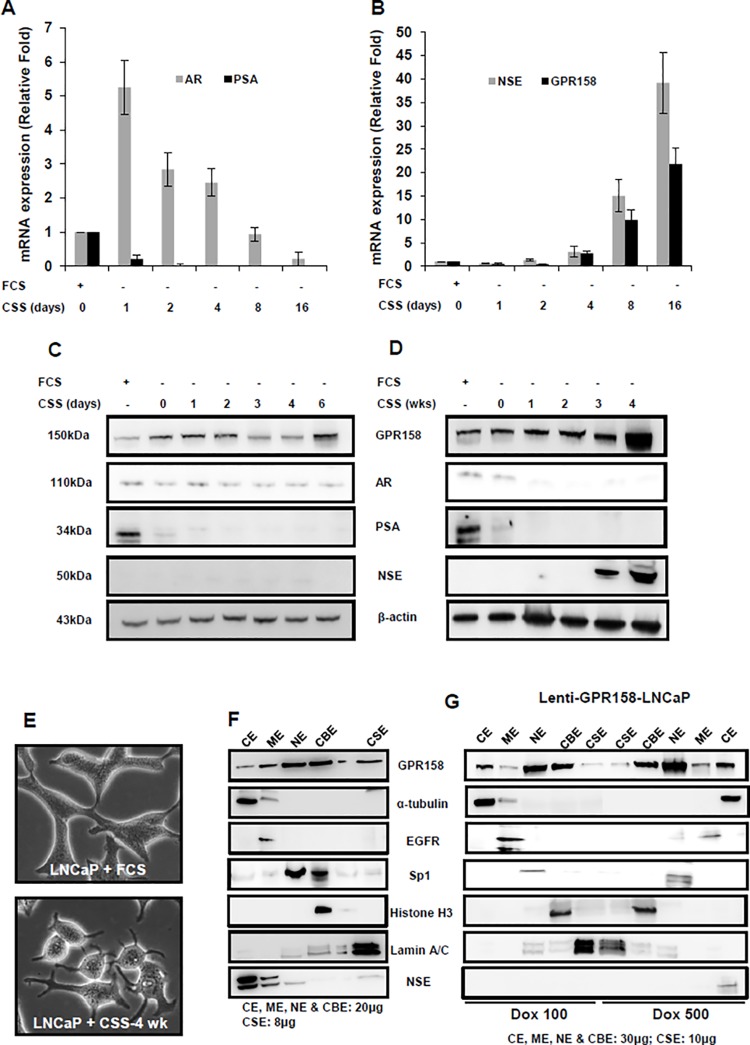
GPR158 expression in NED. **(A, B, C, D)** LNCaP cells were androgen-starved for a short **(A, B, C)** and long **(D)** duration as indicated. Following incubation, total RNA was subjected to qRT-PCR analysis **(A and B)** and whole cell lysates were processed for western blotting **(C and D)** for estimation of GPR158, AR, PSA and NSE mRNA and protein expression levels. The results are representative of two independent experiments performed in duplicate. **(E)** Representative microscopic image of LNCaP cell grown in complete media or in CSS for androgen-starvation for 4-wks is shown. **(F)** LNCaP cells were grown in 10% CSS for 4-wks, leading to NED. **(G)** Lenti-GPR158-LNCaP cells were induced with Dox at 100ng/mL or 500ng/mL for 3 days. **(F and G)** The cells were then subjected to subcellular fractionation as described in Materials and Methods. The loaded amount of protein per fraction is shown. The western blotting was performed using anti-ICD GPR158 antibody. Specific protein markers were used to validate and confirm the purity of the five subcellular fractions examined: cytoplasmic extract (CE) = alpha-tubulin, membrane extract (ME) = EGFR, soluble nuclear extract (NE) = Sp1, chromatin-bound nuclear extract (CBE) = histone H3 and insoluble cytoskeletal extract (CSE) lamin A/C. Image J analysis of protein band intensities was used to estimate the percentage of GPR158 protein present in each fraction in relation to the total amount of protein in all fractions.

In a well-studied cell culture model for NED following ADT, androgen-reduced media is used to induce NE transdifferentiation of androgen-sensitive LNCaP cells, and these cells withdraw from the cell cycle, lose expression of the AR, acquire a neuronal phenotype and express multiple NE markers, including neuron-specific proteins and neuropeptides [45–48]. Applying this model, we cultured LNCaP cells in a reduced androgen medium by replacing 10% FCS with 10% CSS, and followed changes in expression of AR, PSA, NSE and GPR158, over both a short (0–16 days) and long (1–4 wks) time course by RT-PCR and western blotting. The results are shown in Fig. 7. The expression levels of the genes of interest, in LNCaP cells maintained in regular 10% FCS containing media, were considered as 1-fold for comparison.

As expected for an androgen-sensitive cell line, LNCaP cells gradually stopped proliferating following androgen withdrawal (not shown). The level of mRNA for the AR was transiently increased by 5-fold on day one after androgen withdrawal, but then slowly declined and was considerably reduced by day 16 (Fig. 7A). The mRNA for PSA was completely abrogated by day 2. In contrast, the levels of mRNA for both the NE marker NSE, and GPR158, remained unchanged until day 4. However, by day 16, expression of both mRNAs was increased dramatically, NSE by 40-fold, and GPR158 by 20-fold (Fig. 7B). AR and GPR158 protein levels were maintained over the first 6 days of androgen starvation. PSA protein was completely abolished by day 2. NSE protein expression was not detected over the first 6 days (Fig. 7C). However, NSE protein levels increased dramatically by 3 and 4 weeks following androgen starvation (Fig. 7D). We also observed a marked increase in GPR158 protein levels at 4 weeks after androgen starvation (Fig. 7D), which correlated with cell morphologic changes typical of NE transdifferentiation, including cell body compaction and the outgrowth of dendrite-like processes (Fig. 7E).

Since transdifferentiated LNCaP cells showed a higher level of GPR158 expression accompanied by morphological changes involving the cytoskeleton, we also assessed the subcellular distribution of GPR158 in this cell type (Fig. 7F). The pattern was similar to C4-2B cells (Fig. 6) in showing GPR158 distribution across all fractions, however still with ∼60–70% of the total GPR158 protein present in the soluble nuclear and chromatin-bound nuclear fractions.

Next, we investigated whether high-level induction of GPR158 can induce NED using our lenti-GPR158 LNCaP cell line. We compared subcellular distribution of low and high dose GPR158 in this cell line, while also determining whether expression of the NED marker NSE occurred with the high dose. We found that ∼80% of the total GPR158 protein in cells treated with DOX at both 100 and 500 ng/mL was present in the soluble nuclear and chromatin-bound nuclear fractions (Fig. 7G), as seen in the parental LNCaP cells (Fig. 6). Significantly however, we detected expression of the NE marker NSE in the CE fraction of cells treated with DOX at 500 ng/mL, but not 100 ng/mL, suggesting that cells with the higher GPR158 levels were undergoing NED (Fig. 7G).

### GPR158 Effects on Anchorage-Independent Colony Formation

**Fig 8:**
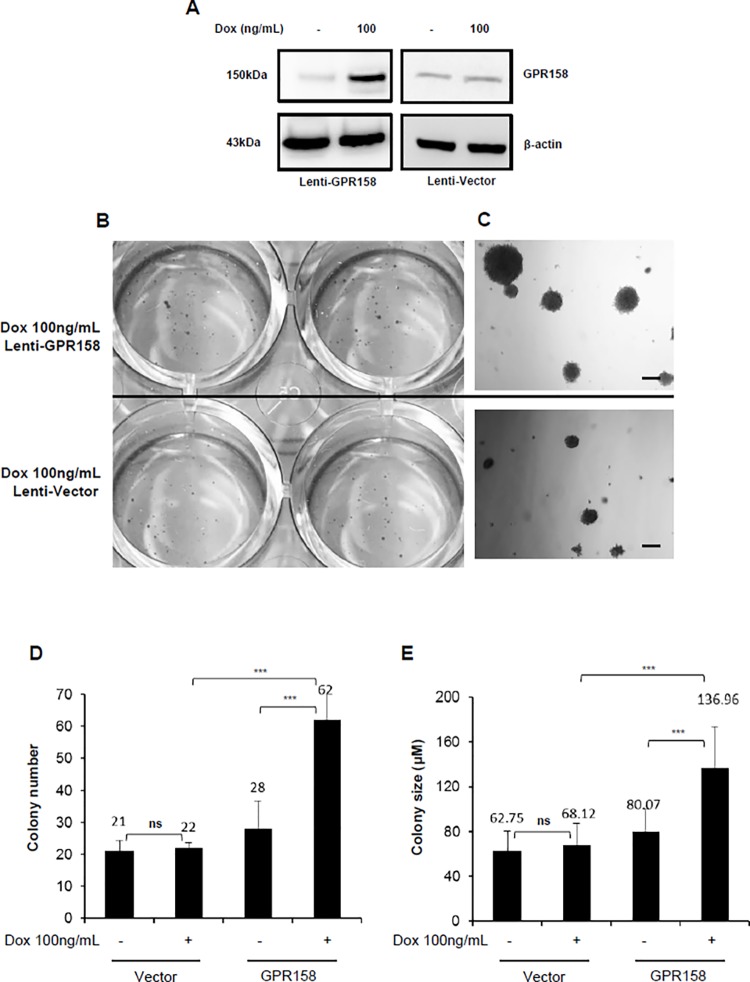
GPR158 effects on anchorage-independent colony formation in LNCaP cells. Soft agar anchorage-independent colony formation assay was performed as described in materials and methods using stably infected lenti-virus encoding either GPR158 (Lenti-GPR158) or vector control (Lenti-vector) LNCaP cells. **(A)** Lenti-GPR158 or lenti-vector LNCaP cells were treated with DOX 100ng/mL for 3-days. The whole cell lysates were subjected to western blot analysis using anti-ICD GPR158 antibodies. **(B and C)** The representative scanned images of culture wells **(B)** and microscopic images under 20x magnification **(C)** of crystal violet stained colonies formed at 14-days post-DOX treatment of lenti-GPR158 or lenti-vector cells are shown. The magnification bar represents 100 μM **(D and E)**. The numbers of colonies were counted under the optical microscope **(D)** and their size was estimated using Nikon Eclipse Ti-E fluorescence microscope software **(E)** for all the indicated sample types. The data represent the mean ± SE of three independent experiments, with each experimental treatment performed in triplicate. ***p<.001; **p<.01, *p<.05; ns, p>.05.

We used our stable lenti-GPR158 LNCaP cell line (and vector control) to ask whether GPR158 over-expression would stimulate anchorage-independent cell colony formation in soft agar medium. GPR158 expression was induced at 100 ng/mL Dox. Results are shown in Fig. 8. We confirmed an induction of GPR158 protein by about 3–4 fold upon Dox treatment for 3-days selectively in lenti-GPR158 LNCaP cells but not in lenti-vector LNCaP cells (Fig. 8A). Lenti-GPR158 and lenti-vector cells (5000 cells/well) were separately seeded in 12-well plates and the cell growth was monitored daily under the microscope for 2 weeks. Our data showed a significant increase in lenti-GPR158 colony formation in both number and size; however, the lenti-vector cells only formed a few small colonies when treated with 100ng/mL Dox every 3-days (p<0.01; Fig. 8, B, C, D and E). The representative scanned images of wells and microscopic images of colonies from Dox treated lenti-GPR158 and lenti-vector are shown in Fig. 8B and C. The average colony number per well, representative of three independent experiments, each performed in triplicate is given as a histogram in Fig. 8D. As compared to Dox-induced lenti-vector, a 3-fold increase in number of colonies was observed from lenti-GPR158 LNCaP cells (Fig. 8D). In addition to increased colony numbers, the size of colonies from lenti-GPR158 LNCaP cells also increased by approximately 2-fold as compared to lenti-vector LNCaP cells (Fig. 8E). Importantly, there was no major significant difference in the number and size of colonies between un-induced lenti-GPR158 and lenti-vector LNCaP cells. These results indicate that GPR158 confers anchorage-independent growth and possibly increase the tumorigenic potential of LNCaP cells.

### GPR158 Expression in Prostate Tumors of the Conditional *Pten* Knockout Mouse

**Fig 9:**
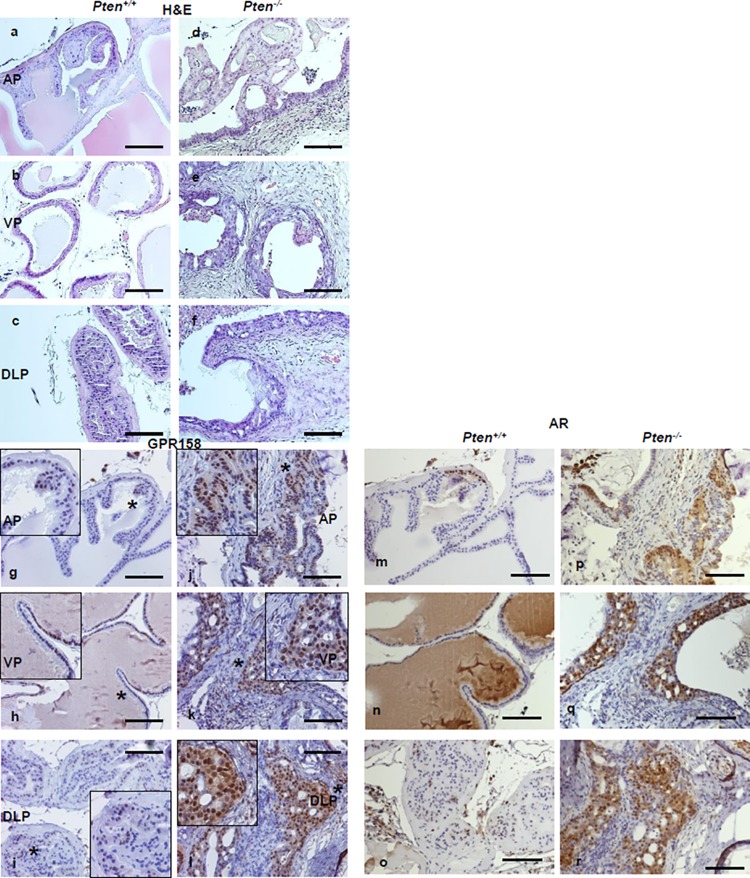
GPR158 expression in prostate tumors of the conditional *Pten* knockout mouse. Representative H&E stain of sections cut from the anterior (AP), ventral (VP) and dorsolateral (DLP) lobes of a 10-month old *Pten* ^*+/+*^ (a-c) or *Pten* ^*-/-*^ (d-f) mouse prostate. IHC localization of GPR158 performed on sections from the *Pten* ^*+/+*^ (g-i) or *Pten* ^*-/-*^ (j-l) mouse prostate. Anti-ICD GPR158 antibody was used at a 1:100 dilution for all the slides. The magnification bar represents 100 μM. The * indicates the region selected for magnified view (g-l). Consecutive sections taken from each lobe from the *Pten* ^*+/+*^ (m-o) or *Pten* ^*-/-*^ (p-r) mouse prostate were immunostained with anti-AR antibodies (1:200 dilution), showing the co-localization of AR and GPR158.

The Pten KO mouse is a well-accepted, genetically defined model for human PCa [37,49]. We performed IHC to localize GPR158 protein in the prostate gland isolated from a 10-month-old conditional *Pten* ^-/-^ mouse and a *Pten* ^*+/+*^ normal littermate control. Our analysis is shown in Fig. 9. In all three prostate lobes: anterior (AP), ventral (VP) and dorsolateral (DLP), the level of GPR158 protein was remarkably higher in tumor tissues of the *Pten* ^-/-^ prostate, as compared with the normal prostate of the *Pten* ^*+/+*^ mouse. Consistent with results of our cell culture studies reported above, GPR158 was predominantly nuclear in epithelial cells, but this differed from lobe to lobe with VP showing relatively higher nuclear localization than AP and DLP in the control *Pten* ^*+/+*^ mouse (g-i). However, all three lobes of the *Pten* ^-/-^ mouse displayed GPR158 expression at a similar level with additional diffuse cytoplasmic staining along with strong nuclear localization (j-l). We observed enhanced GPR158 expression in areas of tumor cell invasion into the surrounding stroma and cells of the invading front showed the most intense staining in all three lobes (j-l), suggestive of *in vivo* contributions of GPR158 in tumorigenicity and invasion of PCa cells. Interestingly, immunostaining for the AR in consecutive sections from all three lobes of the same *Pten* ^*+/+*^ and *Pten* ^-/-^ mouse showed co-localization with GPR158, both found predominantly in cell nucleus (m-o; p-r).

### GPR158 and Disease Free Survival

**Fig 10:**
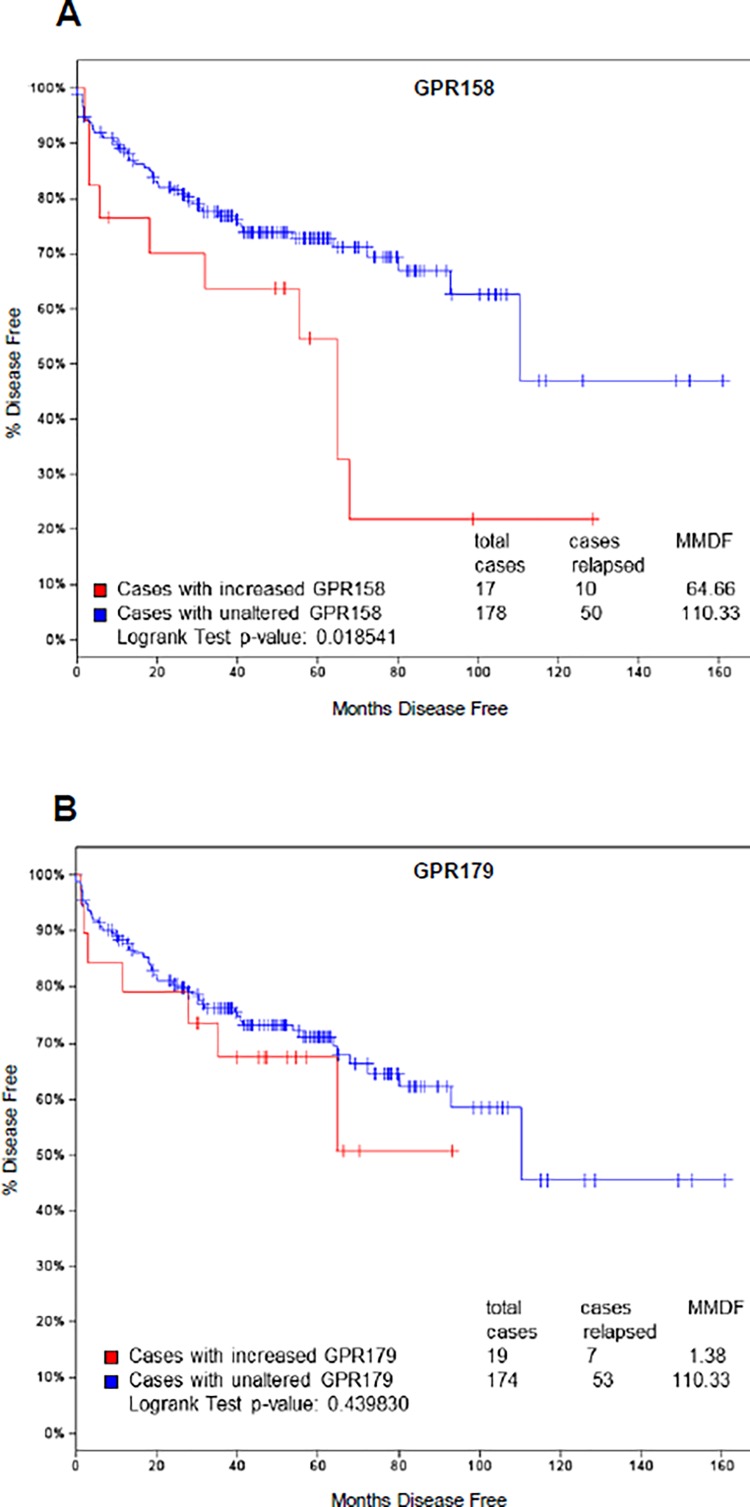
Association of GPR158 levels with lower disease free survival in PCa patients. Kaplan-Meier curves showing percent of patient’s disease free survival *versus* months disease free. The plots were generated using expression analysis of 216 tumors with expression changes of 1.5-fold or higher (the data are taken from [51], analyzed using http://www.cbioportal.org/public-portal/). **(A)** Patients with increased GPR158 mRNA (red line) have lower disease free survival, in comparison with cases of unaltered GPR158 expression (blue line). **(B)** Patients with increased GPR179 showed no change in disease free survival. (**A** and **B**) The numbers of cases used in each group are shown in the graph. The cases with reduced expression of GPR158 (n = 1) and GPR179 (n = 3) were excluded from the analysis. MMDF; median months disease free.

To determine whether GPR158 might be upregulated in human PCa, we searched the cBioPortal, Cancer Genomics database of the Memorial Sloan Kettering Cancer Center [50], and identified a dataset called “Prostate Adenocarcinoma (MSKCC, Cancer Cell 2010) Study” [51]. We selected all tumors (216) as the patient/case set for analysis of GPR158 expression. We found that GPR158 mRNA was altered by 1.5-fold or higher in 9% of all the tumor cases, and it was upregulated in 8% of the cases examined (S4 Fig.). Next, we used the dataset with only increased GPR158 expression at 1.5-fold or higher (18 out of 216 cases) to determine if increased GPR158 expression was associated with disease-free survival of PCa patients. We found that patients with increased GPR158 mRNA expression (red line) have lower rates of disease-free survival (p = 0.018, Fig. 10A), compared to the cases of unaltered GPR158 levels (blue line). Perhaps of significance considering other findings of this study, we found two cases of homozygous PTEN deletion, one case of AR amplification and one case of c-Myc amplification out of 18 cases of upregulated GPR158 expression in PCa patients. Very recently, GPR179, another orphan GPCR in the same family also called GPR158-like, was identified as a possible AR regulator in a genome-wide RNA interference screen [52]. Thus, we also asked whether increased GPR179 mRNA expression (22 out of 216 cases) was similarly associated with disease-free survival in PCa patients. Our analysis showed that increased GPR179 expression was not associated with changes in disease-free survival (p = 0.439; Fig. 10B).

## Discussion

GPCRs comprise very effective molecular targets for pharmacological therapeutics. Many GPCRs show elevated expression in cancers, contributing broadly to tumor growth, progression and metastasis [53], and might serve as therapeutic targets. Many important activities and roles have been identified for specific GPRCs in PCa [54–59]. Results of this study implicate the newly characterized orphan of the glutamate family, GPR158, as another member of the GPCR clan important in PCa. Recently, another glutamate family member, the newly deorphanized GPRC6A, was characterized as a component of a novel NE network regulated by osteocalcin, and evidence for a role in PCa was presented [60]. Importantly however, the findings of the current study suggest that GPR158 acts by mechanisms different than other GPCRs. Moreover, our findings suggest that GPR158 could act at multiple steps in the process of PCa tumorigenesis and progression.

### Evidence that GPR158 Operates via an AR Bypass Pathway

We show that endogenous GPR158 promotes cell proliferation in androgen-sensitive LNCaP cells, but also in three androgen-insensitive PCa cell lines that can proliferate in the absence of androgens. We also observed that GPR158 stimulates expression of the AR in both LNCaP and its androgen-insensitive derivative C4-2B. Clinically, overexpression of the AR is found in up to one-third of CRPC, providing a mechanism to sensitize PCa cells to low serum levels of androgens during ADT [61,62]. Our experiments identify GPR158 as an androgen-responsive gene in androgen-sensitive cells; thus this observation is consistent with a mechanism whereby GPR158 participates in an androgen-regulated positive feedback loop. Androgens are the key regulators of PCa cell proliferation, and early prostate tumors are androgen-dependent. CRPC occurs when tumors evolve to become independent of androgens. However, even in in CRPC, continued AR gene expression and signaling is often seen as critical, as most tumors remain AR-dependent through aberrant mechanisms of AR activation [61]. One of the “outlaw” pathways utilized by GPCRs is activation of receptor tyrosine kinases, leading to ligand-independent activation of the AR, and circumventing the requirement for androgens [53]. This means that GPR158-mediated stimulation of cell proliferation and AR expression could promote growth of both androgen-sensitive and androgen-insensitive tumors. Moreover, endogenous GPR158 promotes cell proliferation in PCa cell lines that are not only androgen-insensitive, but that also appear to lack a functional AR. This suggests that GPR158 must also be able to operate in these cells via a pathway that bypasses the AR altogether.

As mentioned above, the typical GPCR signals through pathways initiated by coupling to heterotrimeric G proteins. In characterized glutamate family members, the long GPCR extracellular domain (ECD) creates a “Venus Flytrap” (VFT) structure that binds amino acids, sugars or ions, thus sensing their presence [3]. This information is transmitted to the 7TM domain, which interacts with heterotrimeric G proteins that control intracellular signaling cascades. GPR158 is different however. While it possesses a long ECD, this domain does not bear features of the VFT. Instead it contains a novel leucine zipper module and a Ca^+2^-binding epidermal growth factor (EGF)-like module, which are typically involved in protein-protein interactions [7,8]. The critical amino acids for coupling with G proteins are conserved in GPR158, thus it is possible that binding of an extracellular protein ligand results in transmission of a canonical signal [7,8]. However, our previous findings in TBM cells from the eye suggest that GPR158 stimulates cell proliferation by a non-canonical mechanism requiring nuclear localization [8]. We show here, that nuclear translocation is also a requirement for GPR158 to stimulate cell proliferation in LNCaP cells.

Our subcellular fractionation experiments identified a high percentage of GPR158 in the soluble nuclear chromatin-bound protein fraction of the nucleus of the four PCa cell lines examined, as well as in normal PHPECs. At this site it would be properly situated to participate in stimulating cell proliferation and AR gene transcription. In our recent publication [8], we noted proteomics studies that identified direct GPR158 binding to transcription factors c-Myc [63] and Pitx2 [64]. Canonical GPCR signaling can enhance expression of the AR via the c-Myc pathway [65]; a role for Pitx2 in regulation of AR expression has also been documented [66]. Thus c-Myc and Pitx2 represent candidates for participating in GPR158-mediated regulation of cell proliferation and AR expression.

IHC data deposited in The Human Protein Atlas (HPA), an on-line database [67], reveals that most normal human organs display weak to moderate nuclear and/or cytoplasmic/membranous positivity for GPR158, primarily in epithelial tissues and glands. A selection of normal prostate and prostatic adenocarcinoma samples included in the database reveal a similar range of distributions, but there is not enough information to determine whether a specific subcellular location of GPR158 might associate with tumorigenesis or progression to CRPC. Our subcellular fractionation studies revealed that a portion of GPR158 protein could also be found in the cytosolic, membrane and cytoskeletal fractions. In particular, the C4-2B sub-line showed considerably more cytoskeletal localization than LNCaP cells, and NED in LNCaP cells was associated with a strong redistribution of GPR158 to the cytoskeletal fraction. Perhaps relevant is a recent comparative genomic and transcriptomic analysis of LNCaP and C4-2B cells that identified 703 differentially expressed genes, with the most significant changes in the ECM-receptor interaction pathways and in focal adhesions [31]. Future studies on GPR158 action at the plasma membrane will be needed to understand the reasons for, and functional implications of, the differential subcellular localization of GPR158 in these cell lines.

### Evidence that GPR158 Plays a Role in NED

An important phenomenon associated with development of CRPC *in vivo* is NED, especially seen with ADT. It has been proposed that NED is normally suppressed by signaling through the androgen receptor [68,69]. PCa tumor growth and NED are at opposite ends of the prostate epithelial cell spectrum of behaviors, with NED characterized by cell cycle withdrawal, loss of AR expression and acquisition of a neuronal phenotype. Here we present evidence that GPR158 could play a part in both PCa tumor growth and NED. One appealing hypothesis for a mechanism to explain this dual role is that, in addition to the documented nuclear activity, GPR158 also has a second activity at the plasma membrane.

While we showed that most of the GPR158 protein continues to localize in the nucleus in NE cells, we observed some subcellular redistribution, with increased GPR158 in the membrane extract, as compared with LNCaP cells. Moreover, total GPR158 increases, thus the amount available at the plasma membrane at any given time would also increase. This could possibly contribute to an overall higher level of plasma membrane activity of GPR158 in NE cells.

As noted above, it is not known whether GPR158 exhibits canonical GPCR activity. However it has been shown that GPR158 can act as a scaffold protein at the plasma membrane, binding the heterotrimeric G protein, Gbeta5, and recruiting members of the R7 family of GTPase Activating Proteins (GAPs), such as RGS7 [7]. In this capacity it functions to inhibit signaling by GPCRs that can interact with R7 family GAPs, i.e., GPCRs that couple with the inhibitory family of Galpha proteins. Significantly, a study that profiled all heterotrimeric G proteins in LNCaP and C4-2B cells found that Gbeta5 is conspicuously absent [70], however, this situation could change with NED. Perhaps of relevance, data mining results obtained in the current study indicate that RGS7 is co-expressed with GPR158 in breast cancer along with many NE markers.

We note that NED in LNCaP cell cultures can be induced in a number of ways, including by elevation of cAMP through treatment with epinephrine, which activates the beta-adrenergic receptor (a GPCR) and stimulates cAMP levels [48,71]. GPR158, acting as a canonical GPCR, might have a similar activity in stimulating cAMP levels. In addition, when acting as a scaffold protein, GPR158 could block the inhibition of adenylyl cyclase mediated by GPCRs that couple with the inhibitory family of Galpha proteins, thus raising intracellular cAMP levels gradually via a positive feedback loop. This intriguing mechanism is currently being investigated.

### Clinical Relevance of GPR158

Our results indicate that increased GPR158 stimulates colony formation in the anchorage-independent soft agar assay, which is considered as an indicator of the capacity for tumor growth *in vivo* [72]. Consistent with this, we found that GPR158 protein expression is highly increased in prostate tumor tissue from all three lobes of prostate isolated from a *Pten* ^*−/−*^ mice, compared with the normal prostate from *Pten* ^*+/+*^ mice. Interestingly, increased GPR158 expression is observed at the leading edges of glands, where cancer cells are invading the surrounding stroma, suggesting that GPR158 up-regulation in *Pten* ^*−/−*^ PCa cells could contribute to tumor invasion.

Most significantly, localization of GPR158 expression corresponded with AR expression. This observation is consistent with the mechanism we proposed based on our cell culture experiments, whereby androgens stimulate expression of GPR158, and GPR158 then stimulates expression of the AR in an androgen-regulated positive feedback loop. Examination of GPR158 expression in NED, and in androgen-insensitive tumors that recur following castration in this mouse model, is now being pursued in a thorough follow-up study.

Importantly, the Kaplan-Meier analysis on the dataset from the Memorial Sloan Kettering cancer genome portal showed that increased GPR158 expression in tumors is associated with lower disease-free survival. Combined with the other findings presented here, this strongly suggests that pharmaceuticals targeting GPR158 could represent a new approach to the prevention and management of PCa. The novel activities of GPR158 in PCa cells, as characterized in this study, suggest that multiple targeting approaches could be used successful.

## Supporting Information

S1 Fig Detection of GPR158 and AR in the DU145 and PC-3 cell lines used in this study. The DU145 and PC-3 sub-line PC-3^AR+^ were originally characterized and maintained in the lab of a co-author on this study (Coetzee). The Western blots used in Fig. 6 were stripped and re-probed for AR protein. As also described in Fig. 6, the amount of protein loaded for each fraction is indicated. Specific protein markers were used to validate and confirm the purity of the five subcellular fractions examined: cytoplasmic extract (CE) = alpha-tubulin, membrane extract (ME) = EGFR, soluble nuclear extract (NE) = Sp1, chromatin-bound nuclear extract (CBE) = histone H3 and insoluble cytoskeletal extract (CSE) lamin A/C. AR protein is detected in PC-3^AR+^ cells, but not in DU145 cells.
(PDF)

S2 Fig Androgen-mediated regulation of GPR158. A search of the NextBio “Pharmaco Atlas” application was performed using “GPR158” as the query. The results revealed 7 independent studies performed using LNCaP cells and all the studies showed increased GPR158 mRNA expression with DHT treatment. The description of selected biosets (1–7) is shown in the graph.
(PDF)

S3 Fig Effect of Dox treatment on expression of GPR158, AR and PSA in LNCaP cells. Parental LNCaP cells were treated with Dox at the indicated concentration for 3-days. The cell lysates were subjected to western blotting using antibodies for GPR158, AR, PSA and beta-actin. The data represent two independent experiments, each performed in duplicate.
(PDF)

S4 Fig GPR158 and GPR179 mRNA expression in human prostate cancer. Oncoplot diagram of GPR158 and GPR179 expression using data from 216 human prostate cancer samples [51] deposited to the Memorial Sloan Kettering cancer genome portal. Percentage of cases with altered GPR158 (upper panel) and GPR179 (lower panel) fold change of 1.5 or greater is shown.
(PDF)
