# Supplementary figures and images for: Expression and Functional Role of Orphan Receptor GPR158 in Prostate Cancer Growth and Progression
Source: PLoS One. 2015 Feb 18;10(2):e0117758. doi: 10.1371/journal.pone.0117758 (PMC4333349; doi:10.1371/journal.pone.0117758)

**Figure S1. Detection of GPR158 and AR in the DU145 and PC-3 cell lines used in this study.**

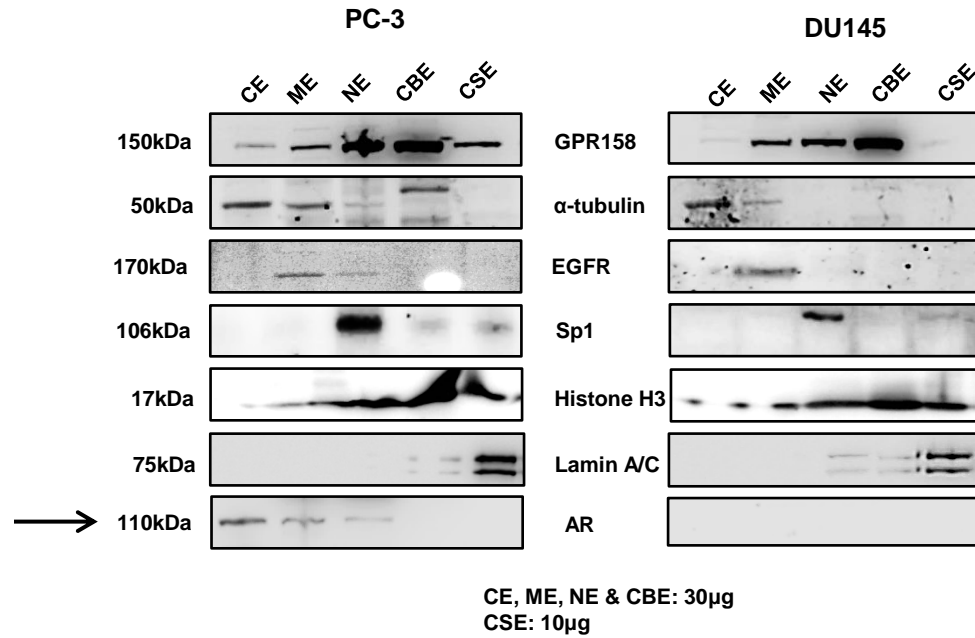

Supplement: S1 Fig — The DU145 and PC-3 sub-line PC-3AR+ were originally characterized and maintained in the lab of a co-author on this study (Coetzee). The Western blots used in Fig. 6 were stripped and re-probed for AR protein. As also described in Fig. 6, the amount of protein loaded for each fraction is indicated. Specific protein markers were used to validate and confirm the purity of the five subcellular fractions examined: cytoplasmic extract (CE) = alpha-tubulin, membrane extract (ME) = EGFR, soluble nuclear extract (NE) = Sp1, chromatin-bound nuclear extract (CBE) = histone H3 and insoluble cytoskeletal extract (CSE) lamin A/C. AR protein is detected in PC-3AR+ cells, but not in DU145 cells. (PDF) [file pone.0117758.s001.pdf]

**Figure S2. Androgen-mediated regulation of GPR158.**

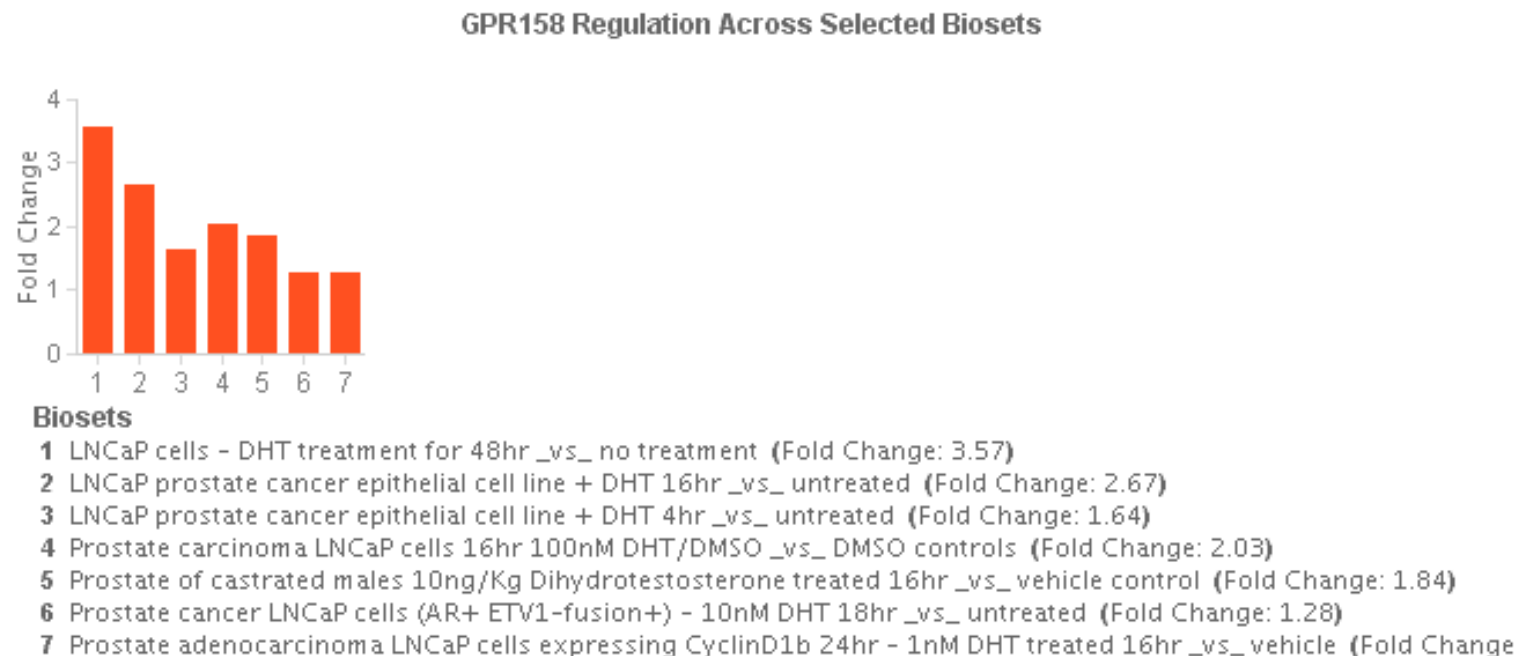

Supplement: S2 Fig — A search of the NextBio “Pharmaco Atlas” application was performed using “GPR158” as the query. The results revealed 7 independent studies performed using LNCaP cells and all the studies showed increased GPR158 mRNA expression with DHT treatment. The description of selected biosets (1–7) is shown in the graph. (PDF) [file pone.0117758.s002.pdf]

**Figure S3.** Effect of Dox treatment on expression of GPR158, AR and PSA in LNCaP cells.

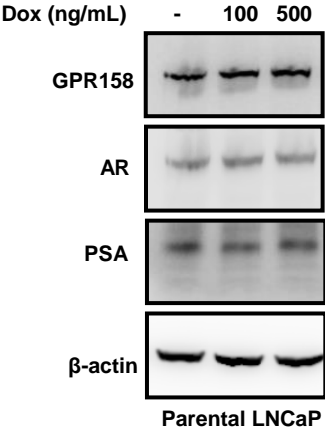

Supplement: S3 Fig — Parental LNCaP cells were treated with Dox at the indicated concentration for 3-days. The cell lysates were subjected to western blotting using antibodies for GPR158, AR, PSA and beta-actin. The data represent two independent experiments, each performed in duplicate. (PDF) [file pone.0117758.s003.pdf]
