# Supplementary material for: Expression and Functional Role of Orphan Receptor GPR158 in Prostate Cancer Growth and Progression
Source: PLoS One. 2015 Feb 18;10(2):e0117758. doi: 10.1371/journal.pone.0117758 (PMC4333349; doi:10.1371/journal.pone.0117758)

Figure S4. GPR158 and GPR179 mRNA expression in human prostate cancer.

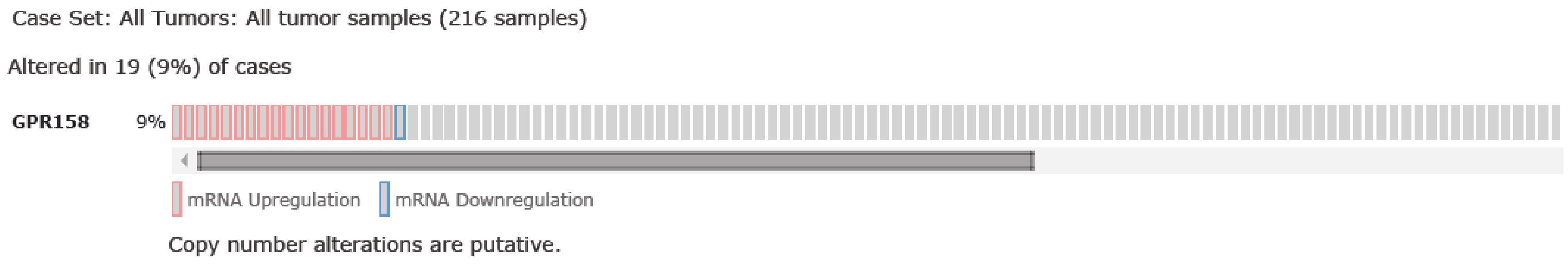

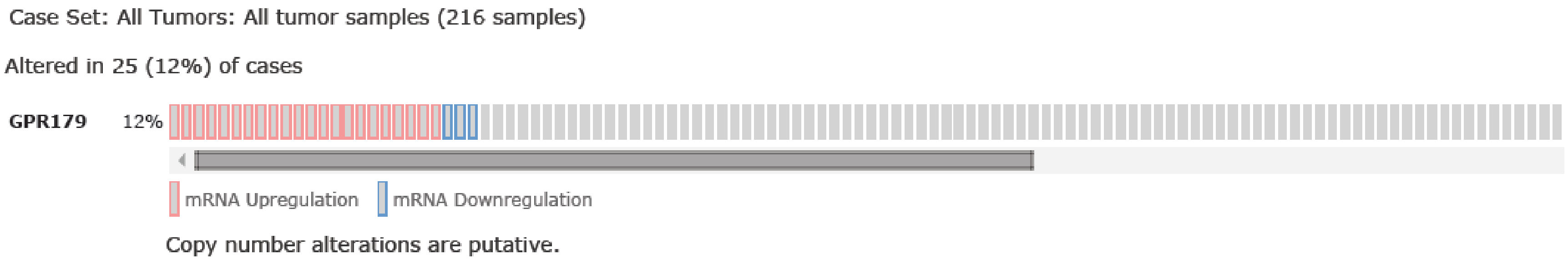

Supplement: S4 Fig — Oncoplot diagram of GPR158 and GPR179 expression using data from 216 human prostate cancer samples [51] deposited to the Memorial Sloan Kettering cancer genome portal. Percentage of cases with altered GPR158 (upper panel) and GPR179 (lower panel) fold change of 1.5 or greater is shown. (PDF) [file pone.0117758.s004.pdf]
